# Systematic analysis of levels of evidence supporting American Academy of Ophthalmology Preferred Practice Pattern guidelines, 2012–2021

**DOI:** 10.1186/s12886-023-02866-9

**Published:** 2023-03-31

**Authors:** Ailin Song, Jay B. Lusk, Anthony N. Kuo, Kelly W. Muir, Sandra S. Stinnett, Durga S. Borkar

**Affiliations:** 1 Duke University School of Medicine, Durham, NC 27705 USA; 2 Duke University Eye Center, Durham, NC USA; 3 Durham Center of Innovation to Accelerate Discovery and Practice Transformation, Durham Veterans Affairs Health Care System, Durham, NC USA

**Keywords:** Preferred Practice Pattern, Guideline, American Academy of Ophthalmology, Level of evidence, Evidence-based medicine, Value-based care, Cost, Value, Randomized controlled trials, Real-world evidence

## Abstract

**Background:**

Despite the increased emphasis on evidence-based medicine, the current state of evidence behind ophthalmology clinical practice guidelines is unknown. The purpose of this systematic analysis was to understand the levels of evidence (LOE) supporting American Academy of Ophthalmology (AAO) Preferred Practice Pattern (PPP) guidelines, assess changes over time, and compare LOE across ophthalmology subspecialties.

**Methods:**

All current PPP guidelines and their immediate predecessors were comprehensively reviewed to identify all recommendations with LOE provided (I [randomized controlled trials], II [case–control or cohort studies], and III [nonanalytic studies]).

**Results:**

Twenty-three out of 24 current PPPs had a prior edition. Among the PPPs with a prior edition, the number of recommendations with LOE decreased from 1254 in prior PPPs to 94 in current PPPs. The number of recommendations with LOE I decreased from 114 to 83, LOE II decreased from 147 to 2, and LOE III decreased from 993 to 9. However, the proportion of LOE I recommendations increased from 9 to 88%, driven by a disproportionate decrease in reporting of evidence lower than LOE I. Subgroup analysis by subspecialty showed similar trends (LOE I recommendations in prior PPPs vs current PPPs: retina: 57 [12%] vs 19 [100%]; cornea: 33 [5%] vs 24 [100%]; glaucoma: 9 [23%] vs 17 [100%]; cataract: 13 [17%] vs 18 [100%]).

**Conclusions:**

Trends in LOE reporting in PPP guidelines indicate an increasing emphasis on evidence from randomized controlled trials from 2012 to 2021. The decline in the number of recommendations with LOE reported suggests an area for improvement in future guidelines as the presence of LOE is crucial to facilitate interpretation of clinical practice guidelines.

## Background

Over the past four decades, there has been an increased emphasis on evidence-based medicine across all medical specialties, particularly in the development of clinical practice guidelines. However, prior analyses of clinical guidelines in cardiology suggest that randomized controlled trials [1], typically considered the highest level of evidence, are not cited in a majority of clinical guidelines developed by key professional societies [2, 3]. Additionally, the proportion of recommendations citing the highest level of evidence has not increased significantly over time [2].

Prior studies in ophthalmology have evaluated the types of evidence published in ophthalmology journals [4]; however, there is limited literature analyzing the levels of evidence present in ophthalmology clinical guidelines. The American Academy of Ophthalmology Preferred Practice Patterns guidelines designate recommended diagnostic and treatment approaches for various ophthalmic conditions and are typically revised every five years [5]. The purpose of this systematic analysis of the American Academy of Ophthalmology Preferred Practice Pattern guidelines was to understand the evidence behind current guidelines, assess changes over time in the levels of evidence used to generate recommendations, and compare levels of evidence utilized across guidelines from different ophthalmology subspecialties.

## Methods

### Review of Guidelines

Current American Academy of Ophthalmology (AAO) Preferred Practice Pattern (PPP) guidelines were identified as those posted on the AAO website (https://www.aao.org/guidelines-browse) as of March 20, 2022. Only full-text PPP guidelines documents were included. Summary Benchmarks and PPP Clinical Questions were not included. Since PPP guidelines are typically valid for five years, the immediate predecessors of current guidelines were identified to assess changes over time. Prior guidelines were either identified on the AAO website (https://www.aaojournal.org/content/preferred-practice-pattern) or requested from the AAO if they were issued prior to 2015*.* No human subjects, human-derived materials, or human medical records were involved in this study to necessitate review by an Institutional Review Board.

The guidelines report levels of evidence (LOE) based on the Scottish Intercollegiate Guidelines Network (SIGN) scale (Table 1) [6]. The guidelines also report quality of evidence and strengths of recommendation defined by the Grading of Recommendations Assessment, Development, and Evaluation (GRADE) scale (Table 1) [7]. Current PPPs report SIGN and GRADE ratings throughout the PPP main texts. Prior PPPs report the ratings in a centralized appendix.

**Table 1:** The Scottish Intercollegiate Guidelines Network (SIGN) scale and the Grading of Recommendations Assessment, Development, and Evaluation (GRADE) scale

**SIGN scale**	**Definition**
I + +	High-quality meta-analyses, systematic reviews of randomized controlled trials (RCTs), or RCTs with a very low risk of bias
I +	Well-conducted meta-analyses, systematic reviews of RCTs, or RCTs with a low risk of bias
I-	Meta-analyses, systematic reviews of RCTs, or RCTs with a high risk of bias
II + +	High-quality systematic reviews of case–control or cohort studies High-quality case–control or cohort studies with a very low risk of confounding or bias and a high probability that the relationship is causal
II +	Well-conducted case–control or cohort studies with a low risk of confounding or bias and a moderate probability that the relationship is causal
II-	Case–control or cohort studies with a high risk of confounding or bias and a significant risk that the relationship is not causal
III	Nonanalytic studies (e.g., case reports, case series)
**GRADE scale**	**Definition**
Good quality	Further research is very unlikely to change our confidence in the estimate of effect
Moderate quality	Further research is likely to have an important impact on our confidence in the estimate of effect and may change the estimate
Insufficient quality	Further research is very likely to have an important impact on our confidence in the estimate of effect and is likely to change the estimate Any estimate of effect is very uncertain
Strong recommendation	Used when the desirable effects of an intervention clearly outweigh the undesirable effects or clearly do not
Discretionary recommendation	Used when the trade-offs are less certain—either because of low-quality evidence or because evidence suggests that desirable and undesirable effects are closely balanced

For each guideline, recommendations with reported LOE were abstracted by one of two reviewers (either A.S. or J.B.L.). Any statement with a reported LOE was considered a recommendation. Further details of LOE reporting are presented in Table 1. For each recommendation with LOE reported, we recorded the LOE, the quality of evidence, and the recommendation strength. Each recommendation was categorized as a recommendation for diagnosis, management, or both. We also recorded the subspecialty associated with each PPP guideline following the subspecialty categories listed on the AAO PPP website (cataract/anterior segment, comprehensive ophthalmology, cornea/external disease, glaucoma, neuro-ophthalmology, ocular pathology/oncology, oculoplastics/orbit, pediatric ophthalmology/strabismus, refractive management/intervention, retina/vitreous, and uveitis).

An additional comprehensive review of all sentences in the Care Process section, where recommendations are typically located, of the 2021 edition of the Cataract in the Adult Eye PPP was performed [8]. A single reviewer (A.S.) abstracted all sentences from the document. Two reviewers (A.S. and J.B.L) independently determined whether each sentence constituted a recommendation statement—a sentence was considered a recommendation statement if it addressed how patients should be diagnosed or managed clinically. For each recommendation statement, the two reviewers examined all references in the document associated with the statement to determine their LOE based on the SIGN scale and recorded the highest LOE (LOE I > LOE II > LOE III). When disagreement existed, the two reviewers had a discussion to reach a consensus decision. Further, a third reviewer (A.N.K.), a board-certified anterior segment ophthalmologist and cataract surgeon, validated all the findings.

All current PPP guidelines were also reviewed to determine whether or not cost-effectiveness or cost/value factors were explicitly mentioned as part of the justification for each recommendation. Additional review of each guideline was performed to determine the presence of cost/value statements, broadly defined as any statement in which cost or value was mentioned, and whether such statements were used to: 1) report a gap in cost/value evidence; 2) highlight economic impact of disease or care; and 3) advocate for cost/value-related issues, consistent with a framework previously described in the cardiology literature [9].

### Data analysis

We calculated the total number of recommendations with LOE reported for each current and prior PPP and calculated the change in number of recommendations over time. The median number of recommendations per guideline was determined. The number of recommendations with reported LOE were also summarized by subspecialty and care process category (diagnosis, management, or both).

Additionally, the numbers and proportions of recommendations classified as LOE I, II, and III among all current and prior PPPs were determined. To further assess differences across subspecialties, we compared the number and proportion of LOE I, II, and III recommendations in current PPPs with those in prior PPPs by subspecialty. We also reported the quality of evidence according to the GRADE scale, stratified by LOE.

For the current Cataract in the Adult Eye PPP, agreement between the two reviewers for whether a sentence constituted a recommendation statement was measured by percent agreement and kappa statistics. If references were provided in the document, the numbers and proportions of validated recommendation statements that were classified as LOE I, II, and III were determined. If recommendation statements also had LOE explicitly reported in the document, we compared the reported LOE with the study team-determined LOE.

To evaluate the role of cost/value in PPPs, the proportion of current PPPs that had any recommendation supported by cost-effectiveness or cost/value considerations was determined. Further analysis was done to assess the proportions of current PPPs that contain statements addressing each of the specific areas related to cost/value considerations.

## Results

### Current PPP guidelines

Overall, LOE from 24 current PPP guidelines published between 2017 and 2021 were abstracted. Across the 24 guidelines, the LOE (SIGN) was provided for 94 recommendations. The median number of recommendations with LOE per guideline was 1.5 (interquartile range [IQR]: 1.0—5.0). 83 (88%) recommendations had LOE I, 2 (2%) had LOE II, and 9 (10%) had LOE III. All LOE II and III recommendations were in the 2017 Refractive Errors and Refractive Surgery PPP.

Among the 94 recommendations with LOE, the vast majority (98%) were for management. The remaining 2% were for diagnosis. The number of guidelines per subspecialty area ranged from 0 to 7 (Table 2).

**Table 2:** Number of recommendations with reported level of evidence (LOE) in current and prior American Academy of Ophthalmology Preferred Practice Pattern guidelines

Guideline by subspecialty^a^	Number of recommendations with a LOE in current guidelines	Number of recommendations with a LOE in prior guidelines	Change in number of recommendations with a LOE from prior to current
Retina/Vitreous	19	485	-466
-Idiopathic epiretinal membrane and vitreomacular traction	2	7	-5
-Age-related macular degeneration	2	103	-101
-Retinal vein occlusions	4	13	-9
-Idiopathic macular hole	3	68	-65
-Posterior vitreous detachment, retinal breaks, and lattice degeneration	1	86	-85
-Diabetic retinopathy	7	197	-190
-Retinal and ophthalmic artery occlusions	0	11	-11
Cataract/Anterior Segment	18	75	-57
-Cataract in the adult eye	18	75	-57
Glaucoma	17	40	-23
-Primary angle-closure disease	4	11	-7
-Primary open-angle glaucoma	12	20	-8
-Primary open-angle glaucoma suspect	1	9	-8
Cornea	24	648	-624
-Conjunctivitis	10	220	-210
-Dry eye syndrome	7	71	-64
-Corneal ectasia	1	47	-46
-Blepharitis	1	71	-70
-Bacterial keratitis	4	104	-100
-Corneal edema and opacification	1	135	-134
Refractive management/intervention	15	0	15
-Refractive errors & refractive surgery	15	0	15
Pediatric Ophthalmology/Strabismus	1	0	1
-Pediatric Eye Evaluations	1	0	1
-Amblyopia	0	0	0
-Esotropia/Exotropia	0	0	0
-Adult Strabismus^b^	0	–	–
Comprehensive Ophthalmology	0	7	-7
-Comprehensive Adult Medical Eye Evaluation	0	7	-7
Vision Rehabilitation	0	0	0

^a^Neuro-ophthalmology, Ocular Pathology/Oncology, Oculoplastics/Orbit, and Uveitis do not have Preferred Practice Pattern guidelines

^b^The adult strabismus Preferred Practice Pattern guideline was published for the first time in 2019

The guidelines also reported recommendation strengths (GRADE) for 104 recommendations. 86 (83%) recommendations were strong recommendations, and 18 (17%) were discretionary recommendations. These recommendations included the 94 recommendations with reported LOE, of which 78 (83%) were strong recommendations, and 16 (17%) were discretionary recommendations. Importantly, aside from indication of recommendation strengths, the current guidelines did not clearly designate statements as recommendations and thus differentiate them from background or evidence synthesis information in the documents.

The 2021 Cataract in the Adult Eye PPP was the guideline with the greatest number of recommendations with reported LOE (*n* = 18). In our systematic review of this guideline, we identified 510 statements that could be considered recommendation statements. The two reviewers both identified 386 (76%) of these recommendation statements independently. Their overall percent agreement was 82%, and the kappa statistic was 0.64. Ninety-five additional statements were identified as recommendation statements by consensus after initially being included by only one reviewer. Twenty-nine statements were additionally considered recommendations by the third reviewer.

We found that 267 (52%) recommendation statements did not have any reference associated with them. For the recommendations that did have references, we reviewed the references and categorized 92 (18%) recommendations as supported by LOE I, 95 (19%) as supported by LOE II, and 56 (11%) recommendations as supported by LOE III. For the 18 statements that had reported LOE, our categorization had 100% agreement with the reported LOE.

### Changes from prior to current guidelines

Twenty-three current PPP guidelines had a prior edition (published between 2012 and 2016) available for comparison. The current Adult Strabismus PPP was published for the first time in 2019. Across the 23 prior PPPs, LOE were provided for 1254 recommendations, compared with 94 (93% decrease) in current editions. Table 2 shows the changes in the number of recommendations with LOE by subspeciality and topic. Overall, the number of recommendations with LOE has decreased for a majority of specialties and topics.

**Fig. 1:**
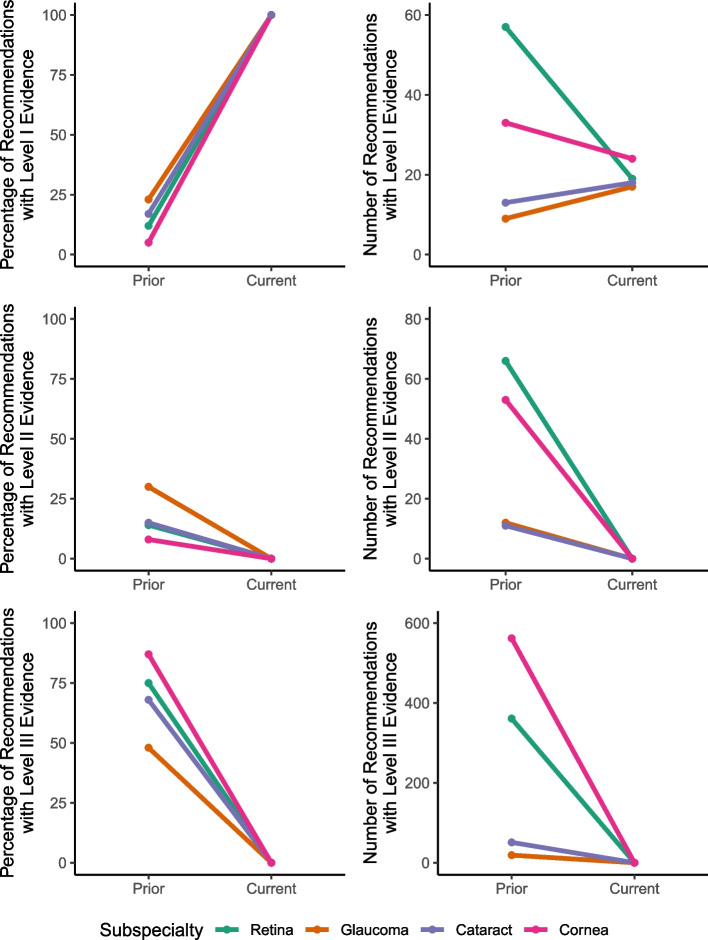
Recommendations with level I, II, and III evidence in current and prior guidelines by subspecialty

Among the 23 PPPs with both prior and current editions available, the number of recommendations with LOE I decreased from 114 to 83, the number of recommendations with LOE II decreased from 147 to 2, and the number of recommendations with LOE III decreased from 993 to 9. The proportion of LOE I recommendations rose from 9 to 88%, driven by a disproportionate decrease in reporting of evidence lower than LOE I. The median number of LOE I recommendations per PPP was 2 (IQR: 0–7), compared with a median of 1.5 LOE I recommendations (IQR: 1.0—5.0) per PPP in current guidelines. A subgroup analysis by subspecialty revealed similar findings (Fig. 1).

**Fig. 2:**
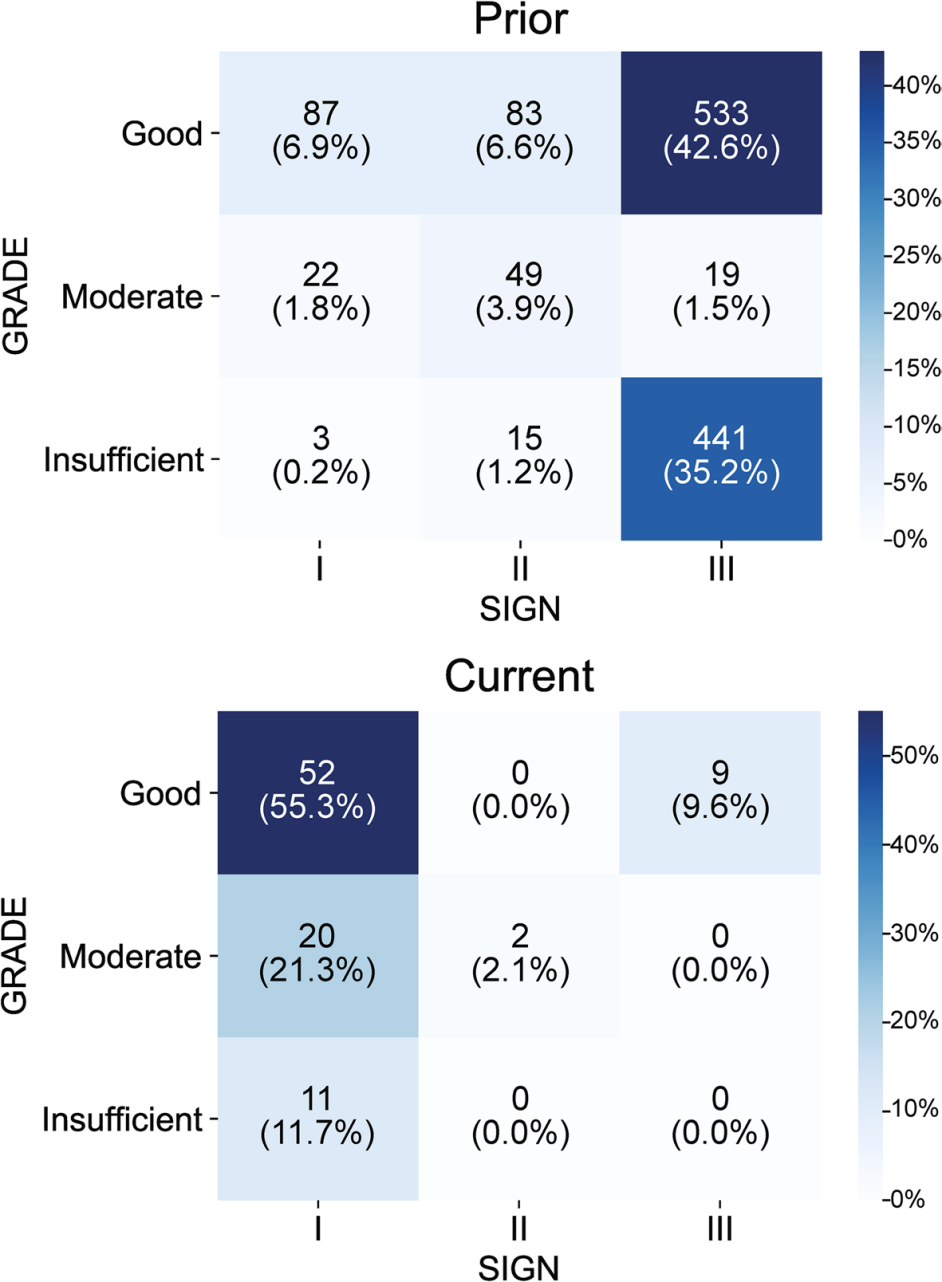
Recommendations by the Scottish Intercollegiate Guidelines Network (SIGN) scale and the Grading of Recommendations Assessment, Development, and Evaluation (GRADE) scale in current and prior American Academy of Ophthalmology Preferred Practice Pattern guidelines

In addition to LOE based on the SIGN scale (LOE I, II and III), both current and prior PPP guidelines reported quality of evidence based on the GRADE scale (good quality, moderate quality, and insufficient quality). Figure 2 shows the proportions of recommendations by level (SIGN) and quality (GRADE) of evidence in current and prior PPP guidelines. Among all recommendations with quality of evidence ratings, current guidelines rated the evidence for 61 (64.9%) recommendations as good quality, 22 (23.4%) as moderate quality, and 11 (11.7%) as insufficient quality. By contrast, prior guidelines rated 761 (57.2%) recommendations as good quality, 109 (8.2%) as moderate quality, and 461 (34.6%) as insufficient quality.

An analysis of all recommendations with insufficient quality evidence in the current guidelines showed that though these recommendations had level I evidence (*n* = 11), the evidence base either had a high risk of bias (i.e., LOE I- based on SIGN) or was not rated for risk of bias. By contrast, in prior guidelines, only 3 recommendations with insufficient quality evidence had level I evidence.

### The role of cost/value in PPP guidelines

**Fig. 3:**
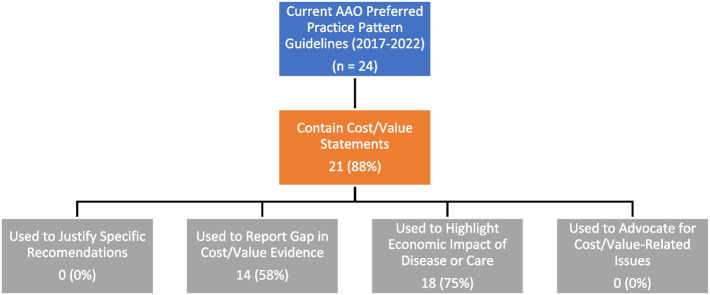
Schema for cost/value statements identified in American Academy of Ophthalmology (AAO) Preferred Practice Pattern guidelines

Among the 24 current PPP guidelines, 21 (88%) guidelines contained cost/value statements. A majority (75%) used cost/value statements to highlight the economic impact of disease or care, and 58% used cost/value statements to report gaps in cost/value evidence. None of them used cost/value considerations to justify specific recommendations or advocated for cost/value-related issues (Fig. 3).

## Discussion

This systematic analysis of LOE supporting AAO PPP guidelines evaluated the proportion of guidelines with a LOE listed, as well as changes in reporting patterns over time and across specialties. Overall, this study demonstrated that while current guidelines report LOE for substantially fewer recommendations, a much higher proportion of recommendations are supported by evidence from randomized controlled trials. Eighty-eight percent of current recommendations with reported LOE had LOE I. Subgroup analysis by subspecialty showed similar trends. These results suggest that while current AAO PPPs emphasize evidence from randomized controlled trials, LOE from other types of studies may not be formally rated or reported.

Although analyses of guidelines have been performed in other specialties, such as cardiology [2, 3], prior investigation of evidence supporting guidelines in ophthalmology is limited. A 2015 study examined the LOE of papers published in four major ophthalmology journals and concluded that lower LOE publications would continue to play a large role in guiding the field of ophthalmology [4]. At first glance, the findings from our study do not appear to suggest this same trend among the reported LOE supporting AAO PPPs, as the vast majority of recommendations with reported LOE had the highest level of evidence.

However, our comprehensive review of the 2021 Cataract in the Adult Eye PPP and independent rating of LOE of the citations show that 30% of recommendations rely on level II and III evidence (vs. 18% level I), but the LOE was simply not reported in the PPP. The majority (52%) of recommendations did not have any citations, consistent with a prior study investigating the relationship between findings from systematic reviews and the 2015 AAO PPP on interventions for age-related macular degeneration [10]. The study found that only 1 out of 35 treatment recommendations in the PPP cited a reliable intervention systematic review [10]. Our study complements the existing literature, highlighting that there may be areas to include additional supporting evidence in AAO PPPs.

In evidence-based medicine, randomized controlled trials (RCTs) and systematic reviews/meta-analyses synthesizing their results are the pinnacle of evidence as randomization reduces bias and allows for investigation of causal relationships. A study conducted in 2019 found that only 2% of all publications in the field of ophthalmology were RCTs [11]. In our study, while the proportion of LOE I (ie, meta-analysis, systematic reviews of RCTs, or RCTs) recommendations has increased from prior PPPs to current PPPs, this increase is primarily driven by a disproportionate underreporting of lower-level evidence. In fact, the number of LOE I recommendations has not increased. On the one hand, this trend suggests guideline authors may have attempted to highlight LOE I recommendations in the current PPPs. On the other hand, fully reporting both level I and lower-level evidence could help to expand the evidence base highlighted in ophthalmology guidelines.

The Institute of Medicine’s landmark reports on clinical practice guidelines were the impetus for the initial development of many guidelines in effect today [12]. In 2011, the Institute of Medicine recommended standards for developing trustworthy clinical practice guidelines [13]. The standards state that for each recommendation in a guideline, “a rating of the level of confidence in the evidence underpinning the recommendation” should be provided [13]. Our results suggest that substantial underreporting of LOE may exist in current PPPs, as the number of recommendations with reported LOE fell from 1254 in prior PPPs to 94 in current PPPs. These results suggest that there is significant opportunity to include level II and III evidence, which, despite risk of bias, is nonetheless often critically important data [14, 15].

Use of data sources such as insurance claims or multi-institutional registries can provide information about real-world clinical practice that cannot be generated by randomized clinical trials [14, 15]. Furthermore, there are clinical questions for which a randomized trial is infeasible, such as for rare conditions or for procedures where shams are not possible, and in these circumstances, lower levels of evidence ought to be weighted more heavily [16, 17]. Since many ophthalmologic diseases have a low incidence and a heavy reliance on surgical management in certain subspecialties, performing randomized controlled trials may be especially challenging [18, 19]. By acknowledging this and including varied levels of evidence in ophthalmology PPPs, authors may be able to more easily adopt the Institute of Medicine’s recommendation about LOE reporting in clinical practice guidelines.

The same report from the Institute of Medicine also proposed that “recommendations should be articulated in a standardized form detailing precisely what the recommendation action is, and under what circumstances it should be performed” [13]. Our findings demonstrated that aside from indication of recommendation strengths (*n* = 104 recommendations across 24 PPPs), the current guidelines do not articulate recommendations in a standardized form.

Without such standardization, our comprehensive review of the 2021 Cataract in the Adult Eye PPP identified 510 statements that addressed how patients should be diagnosed or managed clinically and thus could be considered recommendations. While the agreement between our two reviewers was good (k statistic = 0.64), this result suggests that interpretations of potential recommendation statements in the PPP can be variable. Standardized articulation of recommendations would help clinicians clearly identify recommended actions for clinical practice. For example, American Heart Association guidelines list all recommendations in visually distinctive boxes, which stand out from the surrounding text and include levels of evidence [20]. Clearly articulating recommendations could also facilitate the creation and assessment of programs to improve the quality of care.

As health care usage and expenditure continue to rise in the United States, value-based care has become an increasingly important concept [21]. A recent systematic review found that between 75.7 and 101.2 billion was spent on low-value care in the United States [22]. Clinical practice guidelines play an important role in shaping practice patterns and thus may be well-suited to promote high-value care. In this area, prior work in cardiology has evaluated cost and value considerations in contemporary heart failure clinical guidelines [9]. The study concluded that although most contemporary heart failure guidelines contained cost/value statements, they were rarely used to support clinical guidance recommendations.

In the ophthalmology guidelines, a majority (88%) of PPPs included cost/value statements. In particular, the high economic impact of disease or care was frequently highlighted (75% of PPPs). However, cost/value considerations have yet to be incorporated into the development of specific recommendations, representing an avenue for future work in ophthalmology guideline development. More than half of the PPPs also reported gaps in cost/value evidence—ongoing efforts in the field such as the IRIS® Registry, which includes performance metrics, may facilitate real-world evidence generation in this area and help to provide needed data for guideline development [23].

The strengths of this study include analysis of all PPPs spanning a 10-year period, including all contemporary PPPs and their immediate predecessors. This thorough analysis allowed us to assess evolutions of PPPs over time and trends in all the specialties and topics that PPPs cover. Furthermore, we reported levels of evidence exactly as described in the guidelines. Additional strengths of this study included independent two-party grading, with validation by a board-certified anterior segment specialist, of levels of evidence for our review of the 2021 Cataract in the Adult Eye PPP.

The limitations include the potential underreporting of LOE in current PPPs, which prohibits us from drawing conclusions about all evidence supporting PPPs. This is partially addressed by our comprehensive review of the 2021 Cataract in the Adult Eye PPP, including its references, which suggests substantial underreporting of LOE across all LOE and disproportionate underreporting of lower-level evidence.

In conclusion, we performed a systematic analysis of reported LOE supporting AAO PPP guidelines. Compared with prior PPPs, current PPPs emphasize evidence from randomized controlled trials. While underreporting of LOE across all LOE exists, there appears to be a disproportionate underreporting of lower-level evidence. Future guideline development may consider clearly defining recommendations, explicitly reporting LOE associated with each recommendation, and integrating cost/value considerations in recommendations.

## Data Availability

Preferred Practice Pattern guidelines analyzed during the current study are available on the American Academy of Ophthalmology website, https://www.aaojournal.org/content/preferred-practice-pattern.

## Abbreviations

AAO: American Academy of Ophthalmology
GRADE: Grading of Recommendations Assessment, Development, and Evaluation
LOE: Level of evidence
PPP: Preferred Practice Pattern
RCT: Randomized controlled trial
SIGN: Scottish Intercollegiate Guidelines Network
